# The Role of Autophagy as a Mechanism of Toxicity Induced by Multi-Walled Carbon Nanotubes in Human Lung Cells

**DOI:** 10.3390/ijms16010040

**Published:** 2014-12-23

**Authors:** Tamotsu Tsukahara, Yoshikaszu Matsuda, Hisao Haniu

**Affiliations:** 1 Department of Molecular Pharmacology and Neuroscience, Nagasaki University Graduate School of Biomedical Sciences, 1-14 Bunkyo-machi, Nagasaki 852-8521, Japan; E-Mail: ttamotsu@nagasaki-u.ac.jp; 2 Clinical Pharmacology Educational Center, Nihon Pharmaceutical University, Ina-machi, Saitama 362-0806, Japan; E-Mail: yomatsuda@nichiyaku.ac.jp; 3 Institute for Biomedical Sciences, Interdisciplinary Cluster for Cutting Edge Research, Shinshu University, 3-1-1 Asahi, Matsumoto, Nagano 390-8621, Japan

**Keywords:** autophagy, carbon nanotube, lung cell, cell death

## Abstract

Carbon nanotubes (CNTs) are promising nanomaterials having unique physical and chemical properties, with applications in a variety of fields. In this review, we briefly summarize the intrinsic properties of highly purified multi-walled CNTs (MWCNTs, HTT2800) and their potential hazardous effects on intracellular and extracellular pathways, which alter cellular signaling and impact major cell functions such as differentiation, reactive oxygen species (ROS) production, apoptosis, and autophagy. A recent study suggested that the induction of autophagy by CNTs causes nanotoxicity. Autophagy was recently recognized as a critical cell death pathway, and autophagosome accumulation has been found to be associated with exposure to CNTs. Although autophagy is considered as a cytoprotective process, it is often observed in association with cell death, and the relationship between autophagy and cell death remains unclear. Our recent study suggests that the levels of autophagy-related genes (LC3B) and autophagosome formation are clearly up-regulated, along with an increase in numbers of autophagosome vacuoles. This review highlights the importance of autophagy as an emerging mechanism of CNT toxicity.

## 1. Introduction

Carbon nanotubes (CNTs) have been functionalized to improve their biocompatibility and to promote their interaction with biomolecules [1,2,3,4]. The discovery of CNTs has resulted in significant advances in medicine owing to their unique structure and properties [4,5]. This includes applications such as cellular drug delivery systems and biochemical markers [4,5,6]. *In vitro* toxicology studies have attempted to link CNTs with acute and chronic cellular responses. Several investigations of acute toxicity and pathogenicity of CNTs in cellular and animal models have been conducted. To date, documented physiological changes in cells exposed to CNTs include inflammation [7], apoptosis [8], reactive oxygen species (ROS)-related responses [4,9], and cell differentiation. Previous reports suggest that the hazard risk associated with the use of CNTs is strongly dependent on the relative amount of iron catalyst [4]. However, the mechanism underlying the chronic toxicity caused by multi-walled CNTs (MWCNTs), especially the adverse effects in humans, is relatively unclear. Recent *in vivo* studies have shown that dispersed MWCNTs induce interstitial lung fibrosis without causing persistent lung inflammation after several weeks of exposure [10,11]. These reports suggest that MWCNTs induce pulmonary fibrotic response by directly stimulating lung fibrosis. Furthermore, a recent study suggested that CNTs are emerging as a novel class of autophagy inducers [12]. Autophagy, which was first identified in 1963, is a tightly regulated cellular process that maintains homeostasis [13,14]. Common to nearly all eukaryotes, autophagy is a catabolic process in which intracellular degradation of dysfunctional cellular components or invaders occurs within lysosomes [15]. Recently, a variety of nanomaterials have been reported to induce autophagy [16,17,18]. Additionally, autophagy plays an important role in some human diseases, including lung disease, cancer, neurodegenerative disorders, and viral infections [19].

## 2. CNT Cytotoxicity and Impurities

CNTs have a small aerodynamic diameter and can easily penetrate lung tissue [20]. Several mechanisms of lung disease caused by CNTs have been proposed; one hypothesis is that they act as oxidative stimuli and promote inflammation and DNA damage [21,22,23]. The most important factors suggested to cause CNT-mediated toxicity are the impurities, especially catalytic metal contaminants produced during the preparation and purification processes [24,25]. However, it has been also reported that the presence of metal impurities can cause confusion related to cell toxicity and the resulting health risks associated with use of CNTs. Iron and reactive oxygen species (ROS) are being increasingly recognized as important mediators of cell death [26]. ROS are chemically reactive oxygen-containing molecules that are formed as byproducts during normal metabolism of oxygen. The resulting oxidative damage due to ROS causes damage to DNA, proteins, and lipids and the activation of cell signaling pathways [4,27]. Our previous study focused on the acute toxicity of a vapor-grown CNT, HTT2800, which is a highly purified MWCNT that was prepared using high-temperature treatment [22,28]. Shape of HTT2800 is characterized by a high aspect ratio, a diameter of 100 to 150 nm, and a length of 10 to 20 µm [28]. Since the iron catalyst was removed from HTT2800 in an argon atmosphere, only a very low concentration (<20 ppm) of the iron-based material remained [4]. As expected, quantification of ROS produced with exposure to HTT2800 (up to 30 μg/mL) revealed no indication of oxidative stress [4]. This result suggests that the potential hazard risk associated with the use of CNTs is strongly dependent on its iron catalyst content [4]. However, our previous study also indicated that exposure of a human bronchial epithelial cell line (BEAS-2B) to HTT2800 resulted in the release of inflammatory mediators [4]. The production of the proinflammatory cytokines interleukin (IL)-6 and IL-8, an indication of an inflammatory response, increased following HTT2800 exposure [4]. Damage to the bronchial epithelium is often observed in patients with asthma and other allergic diseases of the airways. IL-6 is linked to allergic responses involving asthma [29], while IL-8 is associated with chronic obstructive pulmonary disease [30,31]. Furthermore, several *in vitro* studies have been performed to evaluate the biological effects of HTT2800. In general, programmed cell death can be classified into three main groups: apoptosis, necrosis, and autophagy. HTT2800 exposure in BEAS-2B cells produced a concentration-dependent cytotoxic response, lactate dehydrogenase (LDH) leakage, and DNA damage [4]. The viability of HTT2800-exposed cells was significantly decreased, and the LDH-leakage rate was increased in a dose-dependent manner [4]. In addition, accumulation of HTT2800 was observed around the nucleus; a balloon-like nuclear morphology, which is typically associated with necrotic cell death, was also observed. Cell apoptosis is also commonly evaluated using a caspase-3 activation assay. Indeed, caspase-3 activation correlated with internucleosomal DNA fragmentation of the BEAS-2B cells; however, HTT2800 did not induce common apoptotic fragmentation of caspase-3 [4]. Furthermore, a caspase-3 inhibitor, z-VAD-FMK, failed to suppress HTT2800-induced cell death [18]. This result further supports the idea that HTT2800 triggered the necrotic effects observed in BEAS-2B cells. These results also suggest that HTT2800 induced the loss of cell membrane integrity as well as enhanced DNA damage and cell toxicity. Taken together, our results imply that the highly purified CNT, HTT2800, was prepared at an adequate concentration to induce acute toxicity.

## 3. Intracellular Uptake of CNT and the Underlying Mechanism 

Although the mechanism of cellular uptake of CNTs may differ depending on its formulation and size, the pathways by which CNTs enter cells and their subsequent intracellular trafficking and distribution remain poorly understood. In general, there are three ways by which CNTs can enter a cell: endocytosis, phagocytosis, and diffusion [32,33]. Endocytosis is an essential cellular process that allows for the maintenance of cellular homeostasis and the uptake of substances [34]. Endocytic pathways have been divided into two types: the internalization of large substances (>0.5 μm), termed “phagocytosis”, and the uptake of fluids and small substances and molecules, termed “pinocytosis” [35]. Phagocytosis, involving vesicular internalization of particulate matter such as bacteria, is an active and highly regulated process that involves specific plasma membrane-surface receptors and a signaling cascade [36]. Phagocytosis of CNTs by macrophages depends on the size, solubility, surface charge, and formulation of the CNTs [37] and the macromolecules are internalized into intracellular compartments [38]. These processes are energy-dependent and are impaired at low temperatures and in low ATP environments [39]. On the other hand, diffusion is an energy-independent, passive process by which the CNTs can diffuse through the cellular membrane. Our previous study indicated that HTT2800 was internalized into BEAS-2B cells [4]. Interestingly, after staining the cell nuclei, accumulation of HTT2800 was observed around the nucleus. 

However, despite evidence of HTT2800 uptake into human lung cells and the central role of these cells in HTT2800-induced lung disease, a limited amount of data is currently available to determine the fate of HTT2800 once inside BEAS-2B cells. 

## 4. CNT and Autophagy or Autophagic Cell Death 

**Figure 1:**
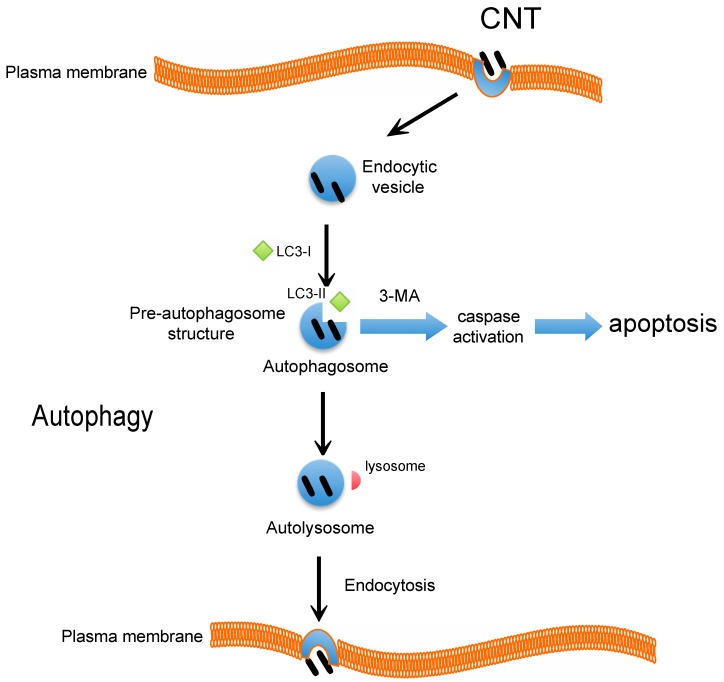
Carbon nanotubes (CNT)-induced autophagy. Schematic representation of the proposed mechanism for HTT2800. During autophagy, a double-layered, membrane-bound autophagosome is formed that surrounds the proteins and damaged organelles identified for degradation. When autophagy is blocked by 3-MA, caspase-3 activation is induced. Autophagy inhibition accelerates apoptosis in cells. Furthermore, HTT2800-induced autophagic cell death occurred in the absence of caspase activation, via an ROS-independent pathway. These results suggest that HTT2800 predominantly causes autophagy rather than apoptotic cell death in BEAS-2B cells.

Autophagosome accumulation induced by CNT treatment was found to be associated with cell death in a majority of published reports [40]. However, the possibility of autophagy inhibition has not been adequately investigated. We recently reported that HTT2800 has emerged as a novel class of autophagy inducers [18]. Autophagy is a tightly regulated cellular process involving bulk cytoplasmic and organelle degradation [41,42,43]. Common to nearly all eukaryotes, autophagy is a lysosomal degradation pathway that recycles intracellular components such as protein aggregates and damaged or dysfunctional intracellular organelles [41]. CNTs have been detected in lysosomes upon internalization, and they have been found to be associated with lysosomal dysfunction [33]. Autophagy was recently recognized as a critical cell death pathway and autophagosome accumulation was found to be associated with exposure to various nanoparticles [17,44]. In eukaryotes, autophagy is the main catabolic mechanism by which the cell degrades cytoplasmic components that are engulfed in double membrane-bound vesicles known as autophagosomes [43]. In the first step of autophagosome development, the cytoplasmic particulate matter is sequestered and then the double-membrane autophagosome fuses with a lysosome to form the autolysosome. To date, three forms of autophagy have been identified: (1) chaperone-mediated autophagy; (2) microautophagy; and (3) macroautophagy, which differ in the mode of delivery to the lysosome [45]. Our recent study suggested that HTT2800-induced autophagy in BEAS-2B cells was based on expression of the autophagic marker, light-chain 3 (LC3) and its effect on cell death and proliferation [18]. This characteristic conversion of LC3 can be used to monitor autophagic activity. LC3 was originally identified as a subunit of microtubule-associated proteins 1A and 1B [46], which associated with the autophagosome membranes after processing. Cleavage of LC3 at the carboxyl terminus occurs immediately following synthesis of the cytosolic form of non-lipidated LC3B-I (19 kDa). During autophagy, LC3-I is converted to lipidated LC3B-II (16 kDa), which is tightly bound to the membrane [46]. HTT2800 significantly induced LC3B-II expression, which decreased after treatment with an autophagy inhibitor, 3-methyladenine (3-MA) [18]. Furthermore, we monitored the cellular distribution of green fluorescent protein-tagged LC3B using fluorescence microscopy. We found that in contrast to the cytoplasmic localization of LC-3B-I, LC3-BII associated with both the outer and inner membranes of the autophagosome and the number of vacuoles was significantly lower in untreated cells than in HTT2800-treated human lung cells [18]. We further examined whether endocytosis and lysosomal regulation might be involved in HTT2800-induced cell death. Prior to HTT2800 exposure, we treated cells with brefeldin A, which inhibits the transport of proteins from the endoplasmic reticulum to the Golgi; 3-MA, which inhibits autophagosome formation; or E64-d and pepstatin A (E64-d + pepstatin A), which are lysosomal proteinase inhibitors. Interestingly, 3-MA and E64-d + pepstatin A, but not brefeldin A, provided protection against the HTT2800-induced cell death that occurred via the autophagic rather than the endocytic pathway [18]. In addition, pretreatment with the caspase inhibitor, z-VAD-FMK, had no significant protective effect on HTT2800-induced inhibition of cell growth. Interestingly, we found that caspase-3 activation was induced in 3-MA-treated BEAS-2B cells [18]. It is also possible that CNTs induce autophagy via an oxidative stress and inflammatory response, such as accumulation of damaged proteins due to a decrease in autophagy flux. It has been reported that the disruption of autophagy flux could lead to enhanced NLRP3 inflammasome accumulation and exaggerated IL-1b production leading to lung fibrosis [47]. These results suggested that autophagy modulation mediated by CNTs may lead to autophagy-mediated cell death and could serve as a specific signal for lung diseases. In conclusion, these studies taken together, suggest that autophagy plays a functional role in the stress response to CNT exposure* in vitro*, and that CNTs might also contribute to autophagic cell death (Figure 1). These markers could serve as possible therapeutic targets that could be explored further in the clinical management of lung diseases.

## 5. Conclusions

Cellular and molecular findings suggest that HTT2800 might be associated with compromised respiratory systems. However, the detailed mechanism of CNT-induced autophagy accumulation in many cases remains unclear. Further studies are needed to elucidate the molecular mechanism underlying the interaction between CNTs and the autophagy machinery. A better-designed prospective study of specific HTT2800-mediated cell regulation would help clarify the mechanisms underlying the link between HTT2800 and cell function.
